# Sugammadex: Appropriate Use in the Context of Budgetary Constraints

**DOI:** 10.1007/s40140-018-0265-6

**Published:** 2018-03-20

**Authors:** Guy Cammu

**Affiliations:** Anesthesiology and Critical Care Medicine, Onze-Lieve-Vrouw Ziekenhuis, Moorselbaan 164, 9300 Aalst, Belgium

**Keywords:** Neostigmine, Neuromuscular block, Pharmacoeconomics, Postoperative complications, Sugammadex

## Abstract

**Purpose of Review:**

The purpose of this review is to assess how sugammadex impacts postoperative residual curarization using appropriate doses based on neuromuscular transmission monitoring and whether the advantages of sugammadex versus neostigmine outweigh its higher cost.

**Recent Findings:**

An accurate assessment of neuromuscular blockade with monitoring is necessary before selecting neostigmine versus sugammadex for reversal at the end of surgery to overcome incomplete neuromuscular recovery. The main advantages of sugammadex over neostigmine are its predictability and its ability to extend the range of blockade reversal. The cost of sugammadex is greater when higher doses of sugammadex are required for antagonism of deep block. Sugammadex probably has the potential to be cost-effective compared with neostigmine if its time savings are put to productive use in clinical practice. However, to date, the economic benefits of the drug are unknown.

**Summary:**

With sugammadex, almost any degree of neuromuscular block can be antagonized within 2–3 min; neostigmine is the only reversal agent effective against benzylisoquinolines and can ideally be used for reversal of lower levels of residual paralysis. The performance of the more expensive sugammadex on improving patient outcomes may depend on several elements of clinical strategy.

## Introduction

The economic benefits of using sugammadex (vs. neostigmine) are unknown. The only paper that considers this matter was very recently published in the British Journal of Anaesthesia and was written by O’Reilly Shah and co-workers. They performed a survey assessing patterns of clinical practice and experience with sugammadex. Of 11,863 anesthesia provider respondents in 183 countries, 5510 (46%) reported that sugammadex was available and relevant to their practice. A majority of these providers (72%) reported selective usage of sugammadex. Most (56%) had some form of extrinsic restriction on sugammadex access primarily due to cost, with far fewer reporting restrictions due to policies or problems with drug availability. Very few were concerned about adverse events (8%). These trends held true among respondents reporting free, unrestricted access to sugammadex [1]. This result was also unexpected, as physician knowledge and awareness of medication costs are generally poor [2] and drug costs generally do not impact individual anesthesia providers directly.

However, the pharmacoeconomics of sugammadex are likely complex, as higher drug costs may be offset by decreased operating room recovery times, faster discharge to the ward and fewer complications related to postoperative residual curarization (PORC) [3]. These means of indirect costs savings, as well as others, may not be fully considered by providers. Moreover, cost issues should not outweigh good clinical practice. The basis of this good clinical practice of managing neuromuscular blockade (NMB) is that for the reversal agent choice, an accurate assessment of degree of NMB with quantitative or objective neuromuscular monitoring is required before the selection of neostigmine versus sugammadex can be made. When selecting a sugammadex or neostigmine dose, the depth of blockade also matters and is equally important.

Most likely, clinicians are interested in a better understanding of the global experience with sugammadex and the impact, if any, of pharmacoeconomics on sugammadex usage. This review is aimed to highlight how sugammadex impacts PORC using appropriate doses of the drug based on quantitative monitoring. The author uses the available literature to determine whether the (theoretical) advantages of the drug versus neostigmine outweigh its higher cost, and consequently, how sugammadex can be potentially used in a cost-effective way.

## PORC

Residual neuromuscular blockade occurs in approximately 20–60% of patients at arrival in the post-anesthesia care unit (PACU) and is associated with an increased incidence of postoperative respiratory complications, such as hypoxemia, pneumonia, and atelectasis and an increased length of stay in the PACU [3–7]. A high incidence of severe residual blockade was observed in patients with critical respiratory events (CREs), which was absent in control patients without CREs [5]. These findings suggest that incomplete neuromuscular recovery is an important contributing factor in the development of adverse respiratory events in the PACU [5]. The incidence of short-term CREs in the PACU is approximately 0.8% [5]. Older age, open abdominal surgery, and duration of operation < 90 min were associated with an increased risk of PORC [8]. The magnitude of the effect of PORC on PACU length of stay is likely to impact patient flow through the operating theater. The economic consequences of PORC-induced delay in PACU discharge are difficult to evaluate and clearly depend on individual institutional factors, including staffing, PACU size, and floor bed availability [7]. More importantly, post-extubation respiratory failure, such as postoperative pneumonia, has been shown to be one of the most significant factors associated with poor patient outcomes, leading to a longer hospital stay and increased financial cost [9]. The occurrence of complications will not only increase hospital costs but also delay return to work and have large implications for society [10].

As residual paralysis plays a primary role in the development of postoperative respiratory morbidity, one could argue that the use of neuromuscular blocking agents (NMBAs) should be avoided. However, the use of a muscle relaxant for tracheal intubation diminishes the incidence of adverse postoperative upper airway symptoms, results in better tracheal intubation conditions, and reduces the rate of adverse hemodynamic events [11]. Moreover, avoidance of NMBAs may increase the risk of difficult tracheal intubation [12]. Nevertheless, the use of NMBAs during anesthesia was associated with an increased risk of clinically meaningful respiratory complications [13], and the incidence of pneumonia in patients receiving a NMBA was 1.8 times that of propensity matched patients who did not receive a NMBA [9]. Another study showed that the use of NMBAs was dose dependently associated with an increased risk of postoperative respiratory complications. Neostigmine was also associated with a dose-dependent increase in pulmonary complications, although exploratory analysis suggested that this result reflected a lack of neostigmine dose adjustment and use of neuromuscular monitoring [14]. Indeed, when given in high doses or unguided by monitoring, neostigmine administration may be associated with an increased incidence of postoperative respiratory complications [15]. However, the proper use of neostigmine guided by neuromuscular monitoring can help eliminate postoperative respiratory complications associated with the use of NMBAs, which was recently re-confirmed in a study where reversal of the effect of muscle relaxants at the end of anesthesia was associated with a decreased risk for postoperative respiratory complications [16]. Finally, another study showed that the incidence of pneumonia in patients receiving a NMBA without reversal of NMB with neostigmine was 2.3 times that of propensity matched cases who received reversal with neostigmine [9].

### PORC in the Elderly

In particular, postoperative pulmonary complications are significantly more common in patients with residual effects of NMBAs who are older than 60 years, which is a steadily growing surgical population worldwide. In a prospective study of 150 elderly and 150 younger patients, the incidence of PORC was high in both groups, but it was higher in the elderly (58% compared with 30%) and was associated with more frequent hypoxemia, postoperative pulmonary complications, and longer hospital length of stay in the elderly [17]. A Scandinavian group has shown that in asymptomatic elderly people, pharyngeal function is often impaired and that the residual effect of a NMBA after general anesthesia worsens the impairment and provides physiological evidence for partial paralysis as a possible cause of postoperative aspiration-induced pneumonia in elderly people [18]. Moreover, PORC in the elderly is attributed to the physiologic changes of aging that alter the pharmacokinetics of NMBAs. Age-related reductions in cardiac output, renal and hepatic function, muscle mass, and ability to regulate temperature are present in most patients who are 70 years old or older [17].

## Appropriate Doses of Sugammadex

Sugammadex rapidly restores neuromuscular function by encapsulating rocuronium. As a result of the one-to-one molecular binding of sugammadex and rocuronium, the necessary dose of sugammadex is dependent on the rocuronium concentration, which can be estimated clinically by neuromuscular monitoring. Accordingly, dose recommendations for sugammadex are based on values obtained by neuromuscular monitoring: reversal of profound rocuronium-induced NMB (i.e., no twitch response after tetanic stimulation), sugammadex 16 mg/kg; reversal of deep NMB [post-tetanic count (PTC) > 1], sugammadex 4 mg/kg; and reversal of moderate NMB [reappearance of the second twitch response (T2) after train-of-four (TOF) stimulation], sugammadex 2 mg/kg. These doses have been shown to restore neuromuscular function in 95% of patients within 5 min [19]. Sugammadex is equally effective at reversing rocuronium-induced block, regardless of whether the maintenance anesthetic regimen is propofol or sevoflurane, whereas sevoflurane reduces the efficacy of neostigmine [20, 21]. One pitfall of using sugammadex is that due to its mechanism of action, under-dosing may lead to a reappearance of NMB after apparent successful recovery [22]. This result teaches us that first, for a reliable reversal of NMB without rebound muscle relaxation, a sufficiently large dose of sugammadex is necessary, and second, in order to assess how much sugammadex is needed, the minimum requirement is a peripheral nerve stimulator [23].

Neostigmine has been used to antagonize the residual effects of NMBAs for decades, but it has the drawback of limited use for deep NMB. Caldwell and co-workers randomized patients to receive sugammadex (4 mg/kg) or neostigmine (70 μg/kg) plus glycopyrrolate at a reappearance of 1–2 PTCs. Mean time to recovery to a TOF ratio of 0.9 with sugammadex was 2.9 versus 50.4 min with neostigmine–glycopyrrolate. Most sugammadex patients (97%) recovered to a TOF ratio of 0.9 within 5 min after administration. In contrast, most neostigmine patients (73%) recovered between 30 and 60 min after administration with 23% requiring more than 60 min to recover to a TOF ratio of 0.9 [24]. Moderate block can be antagonized by anticholinesterase agents as long as sufficient recovery is documented by the presence of at least three responses to TOF stimulation. At this level of block, a full dose of neostigmine (50–70 μg/kg) or sugammadex (2 mg/kg) should be administered. Blobner and co-workers found a lower recovery time variability following reversal with sugammadex when neostigmine or sugammadex was administered at a recovery of T2. This dose resulted in 98% of patients recovering to a TOF ratio of 0.9 within 5 min after sugammadex, representing a rate of recovery that was not achieved until more than 100 min after neostigmine [25]. The message is that in current clinical practice, some patients run the risk of extubation before adequate recovery of the upper airway, especially when neostigmine is used without appropriate neuromuscular monitoring. The high incidence of slow responders after neostigmine may at least partly explain the findings of Grosse-Sundrup and co-workers [13, 26]. A light level of NMB (evidenced by fade to TOF) should be antagonized with lower doses of neostigmine (20 to 30 μg/kg) or sugammadex (1 mg/kg). Routine antagonism of these levels of blocks in all patients with sugammadex is probably economically unreasonable [27••], although neostigmine is not effective in reversing a TOF ratio from 0.2 to ≥ 0.9 within 10 min in 95% patients, whereas sugammadex (0.5 mg/kg) is able to do so [28].

It is not recommended to mix neostigmine and sugammadex for reversal of block for cost-saving reasons as published by Aouad and co-workers, who combined a half-dose of sugammadex with neostigmine for reversal of deep rocuronium NMB [29]. Administering neostigmine together with sugammadex has never been advocated by the manufacturer of sugammadex and is thus off-label per definition. Adding sugammadex after neostigmine may create a situation in which neostigmine induces a weakness. In the study by Aouad and co-workers, it is highly probable that sugammadex removes rocuronium-induced NMB; thus, neostigmine behaves as though no rocuronium has been administered. In that case, muscles are vulnerable to an overabundance of acetylcholine at the neuromuscular junction. Neostigmine administered after full recovery of the NMB with neostigmine-induced depolarizing neuromuscular weakness as a consequence has been extensively described in animals as well as humans [30, 31].

### Cost of NMT Monitoring Devices

Monitoring the effects of NMBAs ensures their appropriate intraoperative use, guides effective antagonism, and helps prevent PORC [32••]. In clinical studies, an association between incomplete neuromuscular recovery and postoperative hypoxemia and respiratory complications has been observed. The use of techniques to limit the degree of residual blockade, such as objective neuromuscular monitoring, may therefore reduce postoperative respiratory impairment and hypoxemia in the early recovery period in the PACU [33]. There is thus ample evidence in the literature indicating that failure to use a simple peripheral nerve stimulator to monitor the degree of paralysis or adequacy of recovery is frequently associated with clinically significant muscle weakness, CREs, and delays in PACU discharge [5, 34]. In addition, neuromuscular monitoring is a necessary hospital cost. Apart from respiratory morbidity, a lack of neuromuscular monitoring significantly increases the risk for distressing awareness during emergence in patients with butyrylcholinesterase deficiency. Indeed, prolonged paralysis after succinylcholine or mivacurium occurs in patients with butyrylcholinesterase deficiency [35].

Moreover, the package insert for sugammadex clearly indicates that the dose of sugammadex should be calculated based on the TOF count. From a pharmacoeconomic perspective, the argument that nerve stimulators are not used because of their high cost makes no economic sense given the use of sugammadex (at a cost of approximately 80€/dose). The approach of administering a certain dose of sugammadex to every patient (regardless of the depth of block) without using a nerve stimulator is of course unreasonable because of the drug’s ineffectiveness in ensuring adequate recovery in all patients [36]. A study demonstrated that the risk of TOF ratio < 0.9 after tracheal extubation after sugammadex remained as high as 9.4% in a clinical setting when neuromuscular monitoring was not used [37].

## Sugammadex and How Much to Pay for It

The reversal activity of sugammadex is selective for steroidal NMBAs. The main advantages of sugammadex over neostigmine are its predictability and its ability to extend the range of NMB reversal. The use of sugammadex is not an excuse to avoid monitoring the depth of blockade. The introduction of sugammadex may present cost challenges. The costs of sugammadex and neostigmine vary among different healthcare systems worldwide. In Belgium, the average cost is 82.7€ for a 200-mg vial of sugammadex, a price that is incomparable to the cost for neostigmine combined with glycopyrrolate (4.05€) (personal communication: Guy Cammu, Onze-Lieve-Vrouw Ziekenhuis, Department of Anesthesiology and Critical Care Medicine, Aalst, Belgium). Neostigmine has been available as a generic for decades, yet its cost in the USA (but not in Europe) has recently increased as a consequence of a Food and Drug Administration approach [38]. The cost of sugammadex is greater when higher doses of sugammadex are required for antagonism of a deep or profound NMB. In a few years, sugammadex will become available as a generic as well, and this change will lead to a lower price. How this change in price will impact its use is unknown. Of note, Raft and co-workers were able to demonstrate that pharmaceutical expenses for anesthesia medication accounted for only 2.4% of the total operating room and PACU cost, even with unrestricted use of sugammadex [39]. For example, the surgical material cost in the author’s hospital for a laparoscopic colectomy amounts to 1929€ (personal communication: Guy Cammu).

Although antagonism of mild residual block with low-dose neostigmine is not quite as prompt as seen with sugammadex, it is probably fast enough in the day-to-day practice of anesthesia. In the present economic environment, it is difficult to propose routine administration of sugammadex in preference to neostigmine when rocuronium-induced neuromuscular recovery has progressed to the point that fade on TOF stimulation can no longer be subjectively detected [40]. For example, the dose of sugammadex required to reverse a TOF ratio of 0.5 was 0.22 mg/kg, which is similar to the characteristics to neostigmine at 34 μg/kg [41]. Based on the efficacy to reverse a TOF ratio of 0.5 in a 70-kg patient, the costs are still approximately 80€ with sugammadex (if one vial is used per patient, even though only 15 mg was used) but approximately 4€ with neostigmine. The sugammadex product characteristics delivered by Merck Sharp & Dohme (MSD), however, state that “After first opening and dilution chemical and physical in-use stability has been demonstrated for 48 hours at 2°C to 25°C, from a microbiological point of view, the diluted product should be used immediately. If not used immediately, in-use storage times and conditions prior to use are the responsibility of the user and would normally not be longer than 24 hours at 2°C to 8°C, unless dilution has taken place in controlled and validated aseptic conditions” [19]. This statement has the potential to allow anesthesiologists to treat their patients’ shallow residual NMB with sugammadex economically as a multi-dose vial. Indeed, in some isolated patients or in some particular clinical circumstances, it may be justified to use sugammadex rather than neostigmine, even for shallow blocks.

### Deep Neuromuscular Blockade During Laparoscopy

Recently, several publications have suggested that intraoperative use of deep NMB, particularly during laparoscopic surgery, could improve surgical conditions. It must be recognized that most studies concluding that deep NMB improves surgical conditions have several flaws in their methodology [42, 43]. Among the most important problems, one can identify the small number of patients, lack of a well-accepted, validated score to grade surgical conditions, and limited number of surgeons involved. To the best of the author’s knowledge, no study has identified that maintaining deep NMB improves surgical outcome or reduces complication rates. The benefits of a sustained deep NMB over a sustained moderate block for laparoscopy are as yet unproven, and multiple costs are associated with keeping NMB deep until the end of the surgical procedure [42]. Large doses of sugammadex (4 mg/kg at a PTC of 1–2) are required at a cost of approximately 160€ per patient. Unfortunately, when deep blockade is present, the time from neostigmine administration to full recovery is over 1 h with very large interindividual variability [44]. The alternative is to mechanically ventilate the lungs until 4 twitch responses return, and then, pharmacologic antagonism with neostigmine can be attempted. This delay also carries a cost, either as time wasted in the operating room or prolonged stay in the PACU [45]. If a TOF count of 1–2 is maintained throughout closure of the fascia, the price of antagonism with sugammadex is already cut in half compared with that of a deep NMB. At a TOF count of 3–4, neostigmine becomes an appropriate antagonist at a fraction of these costs [43].

Even in obese patients, there is not enough good evidence available to justify the routine use of deep NMB, e.g., for laparoscopic bariatric surgery, and the associated important expense of high-dose sugammadex, as recommended dosing by the manufacturer of the drug, is based on real body weight [46]. A randomized, double blind trial by Baete and co-workers found that postoperative pulmonary function substantially decreased after laparoscopic bariatric surgery independent of the NMB regime that was used [47]. More important is that morbidly obese patients are especially susceptible to CREs in the postoperative period, including airway obstruction, hypoventilation, hypercapnia, and hypoxia [48]. The presence of PORC is one of the factors increasing the risk of CREs and should thus be treated with utmost care in this fragile population.

## Cost-Effective Use of Sugammadex…a Gray Zone?

The available evidence from randomized controlled trials suggests that sugammadex produces a substantially faster and more predictable recovery from rocuronium- or vecuronium-induced moderate NMB than neostigmine/glycopyrrolate and can produce a rapid recovery from profound NMB, a facility not available with any other drug. Uncertainties remain concerning its cost-effectiveness [49]. In a systematic review of sugammadex vs. neostigmine that included 17 randomized controlled trials with 1553 participants, sugammadex reduced all signs of PORC and minor respiratory events. There was no difference in CREs. There was no difference in the rate of postoperative nausea or the rate of postoperative vomiting [50]. Sugammadex probably has the potential to be cost-effective compared with neostigmine for the reversal of rocuronium-induced moderate or profound NMB, provided that the time savings are put to productive use in clinical practice, such as by freeing up staff to care for another patient or perform another productive activity. There is also the possibility that extra operations could be scheduled as a result of any reduced recovery time, but again, there is a lack of suitable evidence to support this possibility [49]. It is misleading and incorrect to use cost savings per minute when comparing sugammadex to neostigmine reversal times instead of a proper pharmacoeconomic analysis.

Other issues linked to the advantages of sugammadex vs. neostigmine come from studies that are often not sufficiently powered, have flaws in their protocols, or lack any prospective data to confirm their findings. Examples are as follows:

1. A “cannot intubate, cannot ventilate” event has potentially serious consequences for both patient health and resource use. Although sugammadex is the only reversal agent with the ability to quickly reverse profound block, it remains unclear whether administering sugammadex at 16 mg/kg is cost-effective in such circumstances due to uncertainty over the time it would take to draw up the sugammadex in a high-pressure situation and the lingering effects of hypnotics and opioids also preventing a free airway [51].
2. Well-designed controlled studies are still needed before recommending the routine use of deep NMB and the subsequent routine administration of sugammadex [44].
3. The safe use of sugammadex in patients with neuromuscular disorders enables the anesthetist to perform general anesthesia with NMB in a population in which anesthetists were previously reluctant to use NMBAs and reversal agents. For example, cases are reported in which children with Duchenne muscular dystrophy received a dose of sugammadex to reverse a rocuronium-induced profound NMB. A fast and efficient recovery was achieved, and no adverse events or other safety concerns were observed [52]. It is evident that large series of these kinds of patients and indications are lacking in the literature.
4. EMG of the diaphragm, tidal volume, and PaO_2_ following tracheal extubation were increased after sugammadex compared with neostigmine, reflecting diaphragm-driven inspiration after sugammadex administration. Sugammadex may free more diaphragmatic acetylcholine receptors than neostigmine, although it is unclear whether the full removal of the competing antagonist by sugammadex at the diaphragm reduces the risk of postoperative respiratory complications [53]. To date and to the best of the author’s knowledge, prospective evidence is conflicting as to whether sugammadex reduces postoperative pulmonary complications [54–56].

Finally, it has been shown that once sugammadex is made openly available to providers, anesthesia practice changes, i.e., rocuronium and sugammadex are increasingly used while neostigmine use decreases. This change may impact the cost of anesthesia care, as sugammadex is more expensive. As sugammadex is administered at specific points determined by TOF monitoring, the use of monitoring and the cost for having such a monitor in every anesthesia facility will also increase [49, 57].

## Conclusions

Although numerous anesthetic factors play a primary role in the development of early postoperative CREs, there is a specific role of residual paralysis in the development of postoperative respiratory morbidity. Therefore, appropriate doses of reversal agents (either neostigmine or sugammadex) should always be administered when NMBAs are used, unless full neuromuscular recovery has been documented with quantitative monitoring.

Pharmacologic antagonism, whether using anticholinesterases or sugammadex, must be preferably guided by objective monitoring. Intense and deep levels of NMB cannot be antagonized by anticholinesterases. This depth of block induced by steroidal NMBAs, however, can be reversed rapidly and reliably by the administration of sugammadex, but it requires larger doses of the drug and has associated cost implications. The unrestricted use of sugammadex is likely to increase drug-related costs, and although little doubt exists about the superiority of sugammadex over neostigmine, there is a lack of substantial evidence to suggest that routine use of sugammadex contributes to overall cost savings by means of reducing recovery times in the operating room and PACU. Moreover, an effect on post-PACU outcome or healthcare efficacy has not yet been demonstrated. Nevertheless, sugammadex cost considerations must be balanced in each individual patient and each individual situation against possible advantages that are afforded by the use of sugammadex.
